# Supplementary figures and images for: Understanding the trends, clustering, and risk factors of pinworm infection in preschool settings: A repeated cross-sectional multi-center study between 2019 and 2024
Source: PLoS Negl Trop Dis. 2025 Dec 2;19(12):e0013800. doi: 10.1371/journal.pntd.0013800 (PMC12680333; doi:10.1371/journal.pntd.0013800)

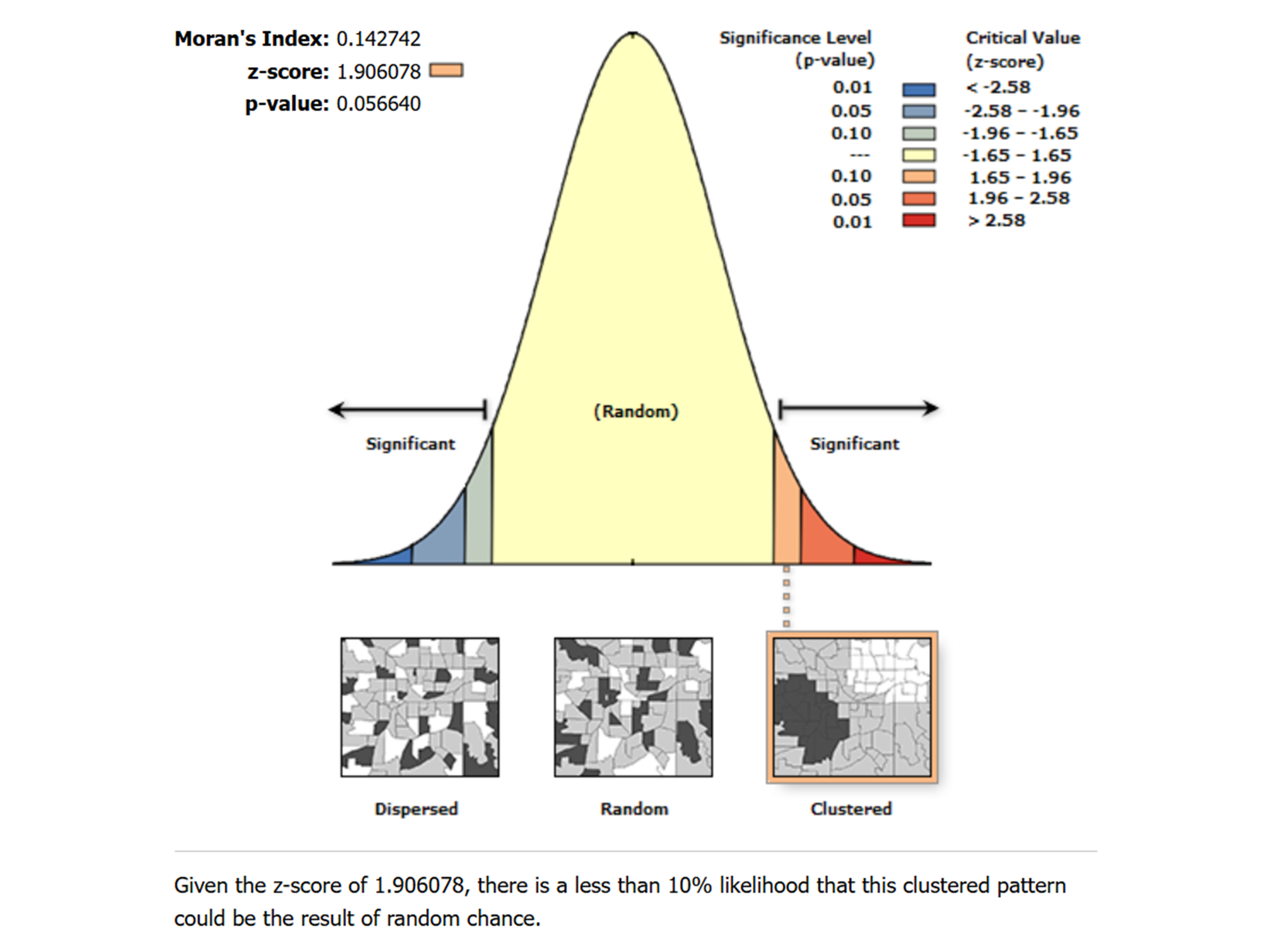

Supplement: S1 Fig — (TIF) [file pntd.0013800.s001.tif]
