# Supplementary material for: Understanding the trends, clustering, and risk factors of pinworm infection in preschool settings: A repeated cross-sectional multi-center study between 2019 and 2024
Source: PLoS Negl Trop Dis. 2025 Dec 2;19(12):e0013800. doi: 10.1371/journal.pntd.0013800 (PMC12680333; doi:10.1371/journal.pntd.0013800)
Supplement: Attachment — Submitted filename: Response to Reviewers.docx [file pntd.0013800.s004.docx]

Dear editors,

On behalf of all the contributing authors, I would like to express our sincere appreciations of your valuable comments concerning our article entitled “Understanding the trends, clustering, and risk factors of pinworm infection in preschool settings: a repeated cross-sectional multi-center study bwteen 2019 and 2024” (PNTD-D-25-01681). We are committed to addressing your comments. Point-by-point responses to the comments are listed below (line number is recorded according to the version with tracked changes).

**Point-by-point responses:**

**Reviewer 1:**

In this manuscript, Mao et al., adopts a 6-year (2019–2024) repeated cross-sectional multi-center design conducted across 45 counties and 104 preschools with a total sample size of 27,925 children in Jiangsu Province, China. This long-term, large-scale framework addresses the scarcity of extended temporal data on pinworm (Enterobius vermicularis) infection in preschool settings. The study also systematically differentiates between rural and urban preschools in sampling, which is also valuable for tailoring location-specific interventions. However, there are still some limitations that can be improved in the future revision.

1) The study collects only one adhesive cellophane tape swab per child, despite noting that single-test sensitivity is ~50% (vs. ~90% for three consecutive daily tests). This likely leads to underestimation of the true infection rate, which could skew trend analyses (e.g., masking subtle fluctuations) or risk factor associations (e.g., diluting the strength of hygiene-related correlations). The authors should discuss this underestimation of the true infection rate.

Response: We appreciate this important comment. We have addressed the limitation under “Limitations” in which we:

(a) explicitly acknowledge that single-test sensitivity is ~50 % and that our prevalence is therefore likely underestimated;

(b) clarify the direction of bias—absolute prevalence is attenuated; risk-factor estimates are shifted toward the null;

(c) recommend repeated sampling for future surveys.

We hope this revision adequately address the concern.

**Line 430-442：**

*Firstly, the sensitivity of one single cellophane tape test is around 50%; however, the sensitivity increases to approximately 90% with tests performed on three different mornings [2]. Consequently, one sample per child in this study prrobably underestimate the true infection rate. This underestimation is expected to attenuate absolute prevalence peaks and bias risk-fator estimates toward the null. Because exposure groups are nondifferentially misclassified, diluting observed associations with hygiene-related variables. Thus, we re-checked all positive and 10% of negative samples to ensure the detection rate. Resource constraints precluded repeated sampling in the present survey; future studies should collect at least three specimens per child to minimise false-negative results and to quantify infection intensity accurately.*

2) While the study identifies migrant status and age as independent risk factors, it provides limited mechanistic explanation for why migrant children are at higher risk. For example, it mentions "family conditions and parental education" but does not analyze whether these factors (e.g., lower parental education, poorer living conditions) mediate the association between migrant status and infection. Could the authors conduct a mediation analysis to assess whether factors like parental education or family income explain the migrant status-infection association?

Response: We thank the reviewer for this insightful suggestion. To clarify the mechanism underlying the higher infection risk in migrant children, we conducted a mediation analysis (SEM with robust MLR) using parental education and family income level as sequential mediators. The results (Table 4) show that parental education and family income level account for 55.2 % of the total effect. The direct effect of migrant status remained significant but was reduced by half, indicating that socioeconomic disadvantages partially mediate the migrant-infection association. These findings have been added to the Results (Socioeconomic pathways mediate the association between migrant status and pinworm infection) and Discussion.

**Line 214-221:**

*To examine the mechanisms underlying the association between migrant status and pinworm infection, we conducted a structural equation model (SEM) with parental education and family income level as parallel mediators. Average education level of both parents and family income level were entered as mediators; infection status was treated as binary. The model was fitted with the WLSMV estimator using the lavaan package. Natural indirect (NIE) and natural direct (NDE) effects were defined and 95 % CIs were obtained with the Delta method. The proportion mediated (NIE/total effect) quantified the magnitude of mediation.*

**Line 339-350:**

***Socioeconomic pathways mediate the association between migrant status and pinworm infection***

*As shown in Table 4, the total effect of migrant status on pinworm infection was 0.540 (95 % CI: 0.420–0.660). The natural indirect effect via parental education and family income level was 0.298 (95 % CI: 0.258–0.337), accounting for 55.2 % of the total effect, whereas the natural direct effect was 0.242 (95 % CI: 0.117–0.368). Parental education and family income level significantly mediated the association. Thus, more than half of the excess infection risk among migrant children is explained by socioeconomic disadvantage.*

***Table 4. Mediation effects of parental education and family income level on the association between migrant status and pinworm infection***

| *Effect path* | *Effect estimate* | *95 % CI* | *Proportion mediated (%)* |
| --- | --- | --- | --- |
| *Natural indirect effect (NIE)* | *0.298* | *0.258–0.337* | *55.2* |
| *Natural direct effect (NDE)* | *0.242* | *0.117–0.368* | *44.8* |
| *Total effect (TE)* | *0.540* | *0.420–0.660* | *100.0* |

**Line 403-419:**

*Parental education and family income level significantly mediated the association, while the direct pathway of migrant status remained significant but was attenuated by almost half, indicating that socioeconomic disadvantage partly explains the higher infection risk among migrant children. This pattern aligns with the social determinants of health framework: parental education shapes health-literacy, hygiene behaviour and health-care seeking, whereas income constrains housing quality, overcrowding and access to preventive products. Consistent with previous parasitology studies, children from lower-income families and with unemployed and less educated mothers showed higher risk of intestinal parasitism (OR=6.0, 95%CI=1.6-22.6; OR=4.5, 95%CI=2.5-8.2; OR=3.3, 95%CI=1.5-7.4 respectively) in rural Mexico[21]. Taken together, the observed mediation suggests that socio-economic disadvantage is not a confounder but a core mechanism; consequently, policies that exclusively target deworming campaigns without improving educational and economic opportunities for migrant families are unlikely to eliminate the excess risk. Integrating conditional cash transfers, preschool subsidies and caregiver health-literacy programmes into the current school-based deworming platform could close 50 % of the infection gap and produce long-term co-benefits for child development.*

**Reviewer 2:**

1.Title: “between 2109-2024” instead of “in 2019-2024”

Response: Thank you for your significant comment. We have revised the “in 2019-2024” to “between 2019 and 2024”.

**Line 1-3:**

*Understanding the trends, clustering, and risk factors of pinworm infection in preschool settings: a repeated cross-sectional multi-center study between 2019 and 2024*

2.The map in figure S1 can be moved to the main text because it is very useful.

Response: We appreciate this helpful suggestion. Following your advice, we have moved the map from Figure S1 to Figure 1 in the main text and updated the legend and in-text citations accordingly.

3.E. vermicularis is not an STH. Please correct this in the abstract.

Response: Thank you for your valuable comment. We have corrected the statement in the abstract.

**Line 26-27:**

*Pinworm (Enterobius vermicularis) remains the most prevalent helminth among preschool-aged children worldwide.*

4.Is E. vermicularis infection also the most prevalent? I doubt. Please support this claim by citing a reliable literature.

Response:Thank you for this valuable comment. We have now softened the original wording and added the following sentence together with up-to-date references in the revised manuscript.

**Line 58-59:**

*Pinworm infection is among the most prevalent helminth infections in young children worldwide, yet we still lack long-term, multi-center data on how it spreads and whether infections cluster within preschools.*

**Line 79-80:**

*Pinworm infection, caused by Enterobius vermicularis, is among the most prevalent helminth infections in young children worldwide [1][2].*

5.Line 120-122: Instead of spring and autumn, specify the months

Response: We appreciate this suggestion. The exact months are now provided as requested.

**Line 136-140:**

*The surveys were conducted once per year, always during one of the two fixed windows: either May-June or from September-October.*

6.Line 147 and elsewhere: Define “raw water”. Is this tap water?

Response: Thank you for pointing this out. To avoid ambiguity we have replaced the term “raw water” with “unboiled tap water” throughout the manuscript.

7.Line 188: correct to “Descriptive statistics are presented”. Correct throughout the results section

Response: Thank you for catching this grammatical error. We have changed the phrase to “Descriptive statistics are presented” throughout the results section.

8.Please specify the guidelines used to set the cut-off for income to low medium and high

Response: Thank you for this helpful comment. We have now added a clear statement in the Variables and measurement section explaining the exact guideline and thresholds used to define low, medium and high family income in the present Chinese setting, as follows:

**Line 167-175:**

*Family income level was classified according to the OECD-modified tercile approach commonly used in Chinese health-equity research (NDRC Macro-team, 2025). Equivalised annual disposable income was calculated by dividing the total household income by the square root of household size. Low income was defined as < 50 % of the national median, medium income as 50–200 % of the national median, and high income as > 200 % of the national median. Based on the 2025 China Household Income Survey, these cut-offs correspond to approximately < ¥90 000, ¥90 000–¥360 000 and > ¥360 000 per year for urban households, and < ¥36 000, ¥36 000–¥144 000 and > ¥144 000 for rural households (CPI-adjusted to the survey year) [16].*

9.Line 204: Figure title should read “Pinworm infection rates in preschools in Jiangsu Province, China, between 2019-2024.” Correct other figure titles accordingly

Response: Thank you for this suggestion. We have revised the figure title to:

“Pinworm infection rates in preschools in Jiangsu Province, China, between 2019 and 2024.”

Analogous formatting corrections have been applied to all table titles to ensure consistency.

*Fig 1. Pinworm infection rates in preschools in Jiangsu Province, China, between 2019 and 2024.*

*Fig 2. Pinworm infection rates in preschools in each city of Jiangsu Province, China, between 2019 and 2024.*

*Table 1. Descriptive statistics of preschool children in Jiangsu Province, China, between 2019 and 2024*

*Table 2. The results of permutation approach quantifying the clustering risk of pinworm infections within a preschool, measured as infection rate ratio (IRR) in Jiangsu Province, China, between 2019 and 2022*

*Table 3. Multivariable stepwise Logistic regression of risk factors for pinworm infection in Jiangsu Province, China, between 2019 and 2024*

10.Figure 1 can be presented using a box plot. The information of both A and B will fit nicely in one box plot (with even more properties of the data, such as median included). This will also allow for the presentation of the rates for in urban vs rural (C). Apply same logic for Figure 2

Response: We have replaced the original charts with box plots that simultaneously display the distribution of infection rates for urban vs. rural settings, thereby providing median, quartiles and outliers in a single panel. The same format has been applied to Figure 2.

**Fig 1. Pinworm infection rates in preschools in Jiangsu Province, China, between 2019 and 2024.** (A) Observed infection rates in individual preschools. (B) Observed infection rates in individual preschools by urban and rural setting.

**Fig 2. Pinworm infection rates in preschools in each city of Jiangsu Province, China, between 2019 and 2024.**

11.Line 288-290: Based on this logic, the variance inflation factor (VIF) for the logistic regression should be reported. The increased risk in infection among migrant children may have been caused by other factors, which could inflate the variance. The VIF will tell whether one factor is affecting another.

Response: We thank the reviewer for this important point. We have now calculated the variance inflation factor (VIF) for each predictor in the final logistic model and added the values in Table A. The highest VIF was 1.055, well below the conventional threshold of 5, suggesting that multicollinearity does not materially inflate the standard errors of our estimates.

**Table A. Variance inflation factors for the multiple stepwise logistic regression model**

| **Variable** | **VIF** |
| --- | --- |
| Year | 1.055 |
| Age | 1.008 |
| Migrant people | 1.047 |

To further clarify the mechanism underlying the higher infection risk among migrant children, we conducted a structural-equation-model-based mediation analysis with parental education and family income level as parallel mediators. The natural indirect effect accounted for 55.2 % of the total effect (NIE = 0.298, 95 % CI 0.258–0.337), while the natural direct effect remained significant (NDE = 0.242, 95 % CI 0.117–0.368). These results indicate that socioeconomic disadvantage largely explains the excess risk of pinworm infection in migrant children and are now reported in the Results.

**Line 214-221:**

*To examine the mechanisms underlying the association between migrant status and pinworm infection, we conducted a structural equation model (SEM) with parental education and family income level as parallel mediators. Average education level of both parents and family income level were entered as mediators; infection status was treated as binary. The model was fitted with the WLSMV estimator using the lavaan package. Natural indirect (NIE) and natural direct (NDE) effects were defined and 95 % CIs were obtained with the Delta method. The proportion mediated (NIE/total effect) quantified the magnitude of mediation.*

**Line 339-350:**

***Socioeconomic pathways mediate the association between migrant status and pinworm infection***

*As shown in Table 4, the total effect of migrant status on pinworm infection was 0.540 (95 % CI: 0.420–0.660). The natural indirect effect via parental education and family income level was 0.298 (95 % CI: 0.258–0.337), accounting for 55.2 % of the total effect, whereas the natural direct effect was 0.242 (95 % CI: 0.117–0.368). Parental education and family income level significantly mediated the association. Thus, more than half of the excess infection risk among migrant children is explained by socioeconomic disadvantage.*

***Table 4. Mediation effects of parental education and family income level on the association between migrant status and pinworm infection***

| *Effect path* | *Effect estimate* | *95 % CI* | *Proportion mediated (%)* |
| --- | --- | --- | --- |
| *Natural indirect effect (NIE)* | *0.298* | *0.258–0.337* | *55.2* |
| *Natural direct effect (NDE)* | *0.242* | *0.117–0.368* | *44.8* |
| *Total effect (TE)* | *0.540* | *0.420–0.660* | *100.0* |

**Line 403-419:**

*Parental education and family income level significantly mediated the association, while the direct pathway of migrant status remained significant but was attenuated by almost half, indicating that socioeconomic disadvantage partly explains the higher infection risk among migrant children. This pattern aligns with the social determinants of health framework: parental education shapes health-literacy, hygiene behaviour and health-care seeking, whereas income constrains housing quality, overcrowding and access to preventive products. Consistent with previous parasitology studies, children from lower-income families and with unemployed and less educated mothers showed higher risk of intestinal parasitism (OR=6.0, 95%CI=1.6-22.6; OR=4.5, 95%CI=2.5-8.2; OR=3.3, 95%CI=1.5-7.4 respectively) in rural Mexico[21]. Taken together, the observed mediation suggests that socio-economic disadvantage is not a confounder but a core mechanism; consequently, policies that exclusively target deworming campaigns without improving educational and economic opportunities for migrant families are unlikely to eliminate the excess risk. Integrating conditional cash transfers, preschool subsidies and caregiver health-literacy programmes into the current school-based deworming platform could close 50 % of the infection gap and produce long-term co-benefits for child development.*

12.Please include title page in the supplementary materials

Response: Thank you for your valuable comment. As suggested, we have incorporated the title page in the supplementary materials.

Thanks again!

Yours sincerely,

Yaobao Liu, PhD.
